# Comparative transcriptome profiling of vanilla (*Vanilla planifolia*) capsule development provides insights of vanillin biosynthesis

**DOI:** 10.1186/s12870-025-06360-w

**Published:** 2025-03-18

**Authors:** Manuel Gastelbondo, Vincent Micheal, Yu Wang, Alan Chambers, Xingbo Wu

**Affiliations:** 1 Plant Breeding Graduate Program, University of Florida, Homestead, FL USA; 2 Environmental Horticulture Department, Tropical Research and Education Center, University of Florida, Homestead, FL USA; 3 Department of Food Science and Human Nutrition, Citrus Research & Education Center, University of Florida, Lake Alfred, FL 33850 USA; 4 Department of Horticulture Sciences, Tropical Research and Education, University of Florida, Homestead, FL USA

**Keywords:** *Vanilla planifolia*, vanillin biosynthesis, transcriptomics, metabolic pathway

## Abstract

**Background:**

Vanillin is the most abundant volatile compound in natural vanilla extract and the primary metabolite from an economic perspective. Natural vanilla is the second most expensive spice in the world and the most profitable crop adapted to the warm tropics. Despite its global popularity, vanilla is mainly cultivated from vegetatively propagated clones and insufficient modern plant breeding has been achieved. One of the breeding objectives is to increase the vanillin concentration in the cured vanilla capsules. The vanillin biosynthesis pathway has not been thoroughly deciphered and multiple hypotheses are considered.

**Results:**

A comparative transcriptomic approach between two accessions with contrasting vanillin content was used to fill in knowledge gaps on vanillin biosynthesis and identify potential candidate genes affecting vanillin accumulation. Out of the 59,128 genes known in vanilla, putative positive and negative regulators that influence vanillin accumulation through pathway modulation, precursor sequestration or enzymatic efficiency were identified. Differentially expressed genes were identified using three specific comparisons on accession, tissue type and developmental stage of capsule. Each comparison was analyzed separately focusing specifically on the accession contrast. BLAST annotation of differentially expressed genes provided protein identities that were mapped to the prominent vanillin biosynthetic pathways proposed by previous studies.

**Conclusion:**

Enzymes from the lignin biosynthetic pathway were found to be negatively correlated to vanillin accumulation in vanilla cured capsules. There were 656 differentially expressed genes shared among all three comparisons and included β-glucosidase, cytochrome P450 and PAL amongst others. These results identify gene targets that could lead to higher vanillin content in vanilla cured capsules.

**Supplementary Information:**

The online version contains supplementary material available at 10.1186/s12870-025-06360-w.

## Introduction

Vanilla is the most popular flavor in the world and natural vanilla extract is the second most expensive spice [1]. Vanilla plants belong to the Orchidaceous plant family which is one of the most diverse plant groups known in the plant kingdom [2]. The orchid sub-family Vanilloideae includes at least 180 recognized species from 15 genera, of which three species from the vanilla genus (*Vanilla planifolia*, *V. pompona* and *V. odorata*) as well as one interspecific hybrid (*V.* x *tahitensis*) are commonly used to produce commercial vanilla extract [3, 4]. Most of the established vanilla plantations around the world cultivate *V. planifolia* (95%), followed by *V.* x *tahitensis* with much smaller contributions from *V. pompona* and *V. odorata* [5, 6]. Vanilla has a unique biology that has attracted studies ranging from ecology, physiology, taxonomy, genetics, nutraceuticals, and food science [4, 7]. As with most orchids, the floral morphology of vanilla prevents spontaneous self-pollination. Natural pollination in vanilla appears at very low rate (< 10%) even in its natural habitat, making hand pollination a necessary process for commercial vanilla production [8]. Newly established *V. planifolia* plantations require four to five years of juvenile growth before transitioning into their reproductive stage. The pollinated flower will develop into a seed-bearing capsule colloquially referred as “bean” or “pod”. Vanilla capsules require eight to nine months after pollination to ripen [9] and are valued based on size, vanillin content and physical integrity. Split, small, and mechanically damaged capsules can still be used to produce vanilla extract at a lower price [10].

Vanilla capsules must undergo a complex curing process to release their aromatic properties. The curing process varies depend on production area but generally consisted of four steps including (1) killing, which prevents capsules from splitting and serves as a partial disinfection step, (2) sweating, which stimulate the enzymatic processes that catalyzes the synthesis of the flavor metabolites, (3) drying, which decrease water content of the capsules, and (4) conditioning, which enhances the flavor by aging, allowing off-flavors to volatilize, and extending shelf life [4]. More than 200 compounds have been identified in natural vanilla extract which offers a delicate aroma profile that cannot be duplicated by synthetic substitutes. Most of the volatile compounds present in natural vanilla extract are categorized as acids, alcohols, ethers, acetals, heterocyclics, phenols, carbonyls, and hydrocarbons [11]. The non-volatile compounds consist of polyphenols, tannins, free amino acids, and resins, which play an important role in retaining the volatile compounds and enhancing the aroma’s longevity. The most abundant aroma-related compound in green vanilla capsules is glucovanillin, which is converted into vanillin (4-hydroxy-3-methoxybenzaldehyde) later in the curing process [11]. Although the metabolite profile of vanilla extract is very complex, vanillin is the major flavor compound and predominantly used to determine the value of vanilla capsules in trade [10]. Despite its wide genetic diversity and phenotypic variations, vanilla is primarily vegetatively propagated in commercial production and has not undergone genetic improvement [1]. Vanilla breeding is a lengthy process given its protracted juvenile development phase. Marker assisted selection has proven to be an effective approach to improve breeding efficiency, but it has not been implemented in vanilla. Molecular marker applications in vanilla have so far been used primarily for diversity analyses and species differentiation [1, 12–15]. Molecular markers associated with key traits such as vanillin content are crucial for new cultivar development [16]. Understanding the genes involved in vanillin biosynthesis is imperative for developing molecular markers to improve the vanillin content through breeding.

Multiple routes have been proposed to describe the vanillin biosynthetic pathway with little consensus (Supplementary Figure S1). Most of the evidence fostered for such proposals has integrated precursor radiolabeling, proteomics and enzymatic assays [10, 17–23]. Though it is agreed that vanillin derives from phenylalanine as a branching point of the shikimic acid pathway, the steps of the proposed pathways differ between these studies. Oxidation was considered as the mechanism for shortening the side chain of phenylpropanoid in one hypothesis [18], while other propositions invoke a non-oxidative process facilitated by 4-hydroxycinnamoyl-CoA ligase (4CL) [18, 21]. Various precursors, including coniferin [22], ferulic acid [22] and glucovanillin [24] are considered for vanillin biosynthesis, yet a definitive outcome remains elusive. The cysteine protease-like protein (CPLP) and phenylalanine ammonia-lyase (PAL) genes were proposed to be key genes controlling the biosynthesis pathway but have yet to be confirmed [19, 21, 23]. Rao et al. (2014) conducted a transcriptome study to identify the key genes involved in vanilla capsule development and identified the majority of monolignol biosynthetic genes responsible for the C-lignin formation in the seed coat of vanilla capsules [25], yet key genes responsible for the regulation of glucovanillin accumulation or glucovanillin-vanillin conversion in the curing process remain unclear.

Molecular markers linked to key genes regulating vanillin biosynthesis can significantly accelerate the vanilla breeding process. The rapid advancement of sequencing technologies has benefited many crops, including vanilla, enabling the creation of genomic resources that facilitate in-depth studies of biosynthesis pathways using omics approaches [12, 26]. In this study, we conducted a comparative transcriptome study on two vanilla accessions with contrasting vanillin content at three development stages and in two different tissues. This analysis provided valuable insights into vanillin biosynthesis, and the candidate genes identified in this study could play a crucial role in developing molecular markers for breeding elite vanilla cultivars.

## Materials and methods

### Plant material and sample preparation

Seven *V. planifolia* accessions (namely AC7, AC94, AC23, AC113, AC67, AC85 and AC110) were selected from the vanilla germplasm collection maintained at the University of Florida’s Tropical Research and Educational Center in Homestead, Florida, USA. Each accession was planted in 20 m^2^ beds at the density of 2 plant/m^2^ using wood mulch as substrate supplemented with monthly foliar fertilizer (Keyplex Inc. FL, USA). The beds were covered with 70% shade and irrigated with overhead sprinklers.

**Fig. 1:**
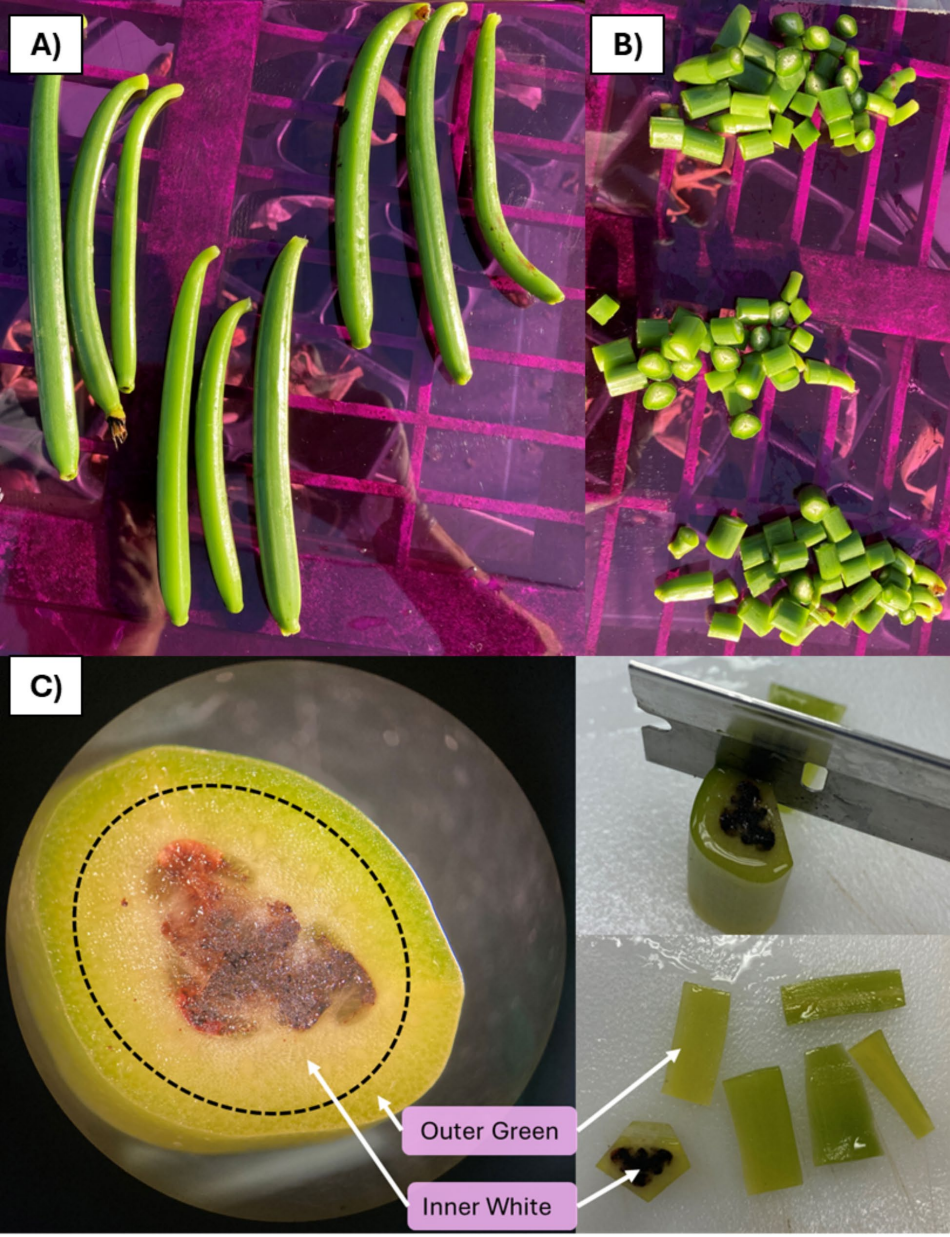
Diagram of vanilla capsule dissection for transcriptomic study. (**A**) Three vanilla green capsules per time point. (**B**) Vanilla capsule in 1 cm x 1 cm fragments. (**C**) Transversal capsule section and tissue dissection

Floral induction of *V. planifolia* was observed in March 2022. The floral racemes were monitored weekly, and fully open flowers were hand pollinated. For each accession, three capsules were harvested at six, seven and eight months after pollination (MAP) (Fig. 1A). Each capsule was divided into 1 cm^2^ fragments (Fig. 1B). Half of the fragments were dissected into two tissue types, the outer green tissue (epicarp and a small residual portion of the mesocarp) and the inner white tissue (mesocarp, endocarp, placenta, and seed tissue), with three replicates per time point (Fig. 1C). The dissected tissue groups were kept at -80 °C for RNA extraction. The second half of the tissue representing three time points was bulked for vanillin quantification. The bulked capsules were subjected to a three minute “kill” step at 63 °C while completely submerged in a water bath inside a plastic zip-lock bag, followed by 72 h incubation at 50 °C inside a glass jar. Finally, the capsule fragments were dried in a vented oven at 32 °C until to reach 25% moisture.

### Vanillin quantification

The relative vanillin content was determined using high-performance liquid chromatography (HPLC) coupled with Ultraviolet–visible detector for seven vanilla accessions. The cured vanilla capsule fragments were subjected for extraction using ethanolic maceration in a 45% ethanol solution at a ratio of 1:10 cured capsule tissue (mass) to ethanol solution (volume). The vanilla extract samples were filtered using a 0.2 μm nylon filter and loaded into the HPLC coupled to a diode array detector (Agilent 1220 Infinity II LC) for measurement. The relative abundance of vanillin was used to estimate the vanillin content. Vanillin and glucovanillin standard curves were generated using synthetic standards (Alfa Aesar A11169 and BioSynth MG66011) for abundance quantification for each sample. The separation was carried out using a Zorbax Extend-C18 analytical column (4.6 mm x 250 mm 5-Micron 80 A) maintained at 30 ℃ in a Hitachi Elite LaChrom column oven L-2350 (Hitachi, Japan). The mobile phase was composed of Solution A: 92.9% Water with 0.1% Triflouroacetic Acid (Fisher HB513), 7% Acetonitrile (Fisher A998), and 0.1% Glacial Acetic Acid (Fisher A38), and Solution B: 99.9% Acetonitrile (Fisher A998) and 0.1% Glacial Acetic Acid (Fisher A38). The gradient elution program was as follows: 0 min, 100% Solution A; 7 min, 98% Solution A, 2% Solution B; 14 min, 60% Solution A, 40% Solution B; 16 min, 59% Solution A, 41% Solution B; 17 min, 100% Solution B terminating at 22 min. UV detection was performed at 272 nm. The injection volume for each sample was 3 µL. The flow rate was set at 1.2 mL/min.

Further vanillin content quantification of the identified vanilla accessions for transcriptome study were completed using Liquid Chromatography with tandem mass spectrometry (LC MS/MS). The cured capsules were cut into 2 cm portions and grinded with a coffee grinder. A total of 5 mg ground capsule powder was used for vanillin extraction in 980 uL of solvent (MeOH/ACN/Water 4:4:2; all LC-MS grade). Vanillin (Ring-^13^C_6_) was used as internal standard by adding 20 uL of 10 mg/L solution for a final concentration of 0.2 mg/L. Samples were vortexed for 5 min and decanted using a centrifuge at 4 ℃ for 15 min at 20,000 ×g. Supernatant was filtered using a 0.20 μm Nylon membrane syringe filter. A standard curve was generated for quantification with 8 levels (90 mg/L– 0.703 mg/L, 2X serial dilution). A 100 µL aliquot of filtered sample was then transferred into a LC vial with a 150 µL insert. Samples were loaded and run in a Thermo Ultimate 3000 HPLC coupled to a Thermo TSQ Quantiva triple-quadrupole electrospray ionization mass spectrometer in tandem (Thermo Scientific, Waltham, MA, USA). A Gemini 3 μm C18 110 Å (150 * 3 mm, 3 μm) column was used at a column temperature of 40 ℃. Two mobile phases (0.1% formic acid in water (phase A) and 0.1% formic acid in ACN (phase B)) were used in a gradient program of 0–8 min; 2–95% B, 8–13 min; 95% B, 13–14 min, 95 − 2% B,14–19 min; 2% B at a flow rate of 0.4 mL/min. The injection volume was set at 5 uL with the following conditions: Static Spray Voltage; Positive Ion 3,500 V, Sheath Gas 35 Arb, Aux Gas 10 Arb, Sweep Gas 0 Arb, Ion Transfer Tube Temperature 325 ℃, Vaporizer Temperature 275 ℃, Dwell Time 50 ms and CID gas 2 mTorr using positive ion mode.

### RNA extraction and sequencing

Total RNA was extracted using the Omega Bio-tek E.Z.N.A.^®^ Plant RNA Kit (Norcross, Georgia, USA) with an on-column DNase treatment. The RNA concentration and quality was determined using the Qubit^®^ broad RNA assay kit [26]. The cDNA sequencing libraries were generated using True-Seq™ RNA Library Prep Kit (Illumina CA, USA) following the manufacturer’s instructions and sequenced on an Illumina NovaSeq X platform (Novogene, Sacramento, CA, USA). A total of 36 samples, representing three time points, two tissue types, and two accessions with three biological replicates each, were used to capture expressing transcripts (Table 1).

**Table 1:** List of samples with three biological replicates for transcriptome study

SampleID	SampleName	Accession	Time points	Tissue type
M01	AC23M6GREEN	AC23	M6	GREEN
M02	AC23M6GREEN	AC23	M6	GREEN
M03	AC23M6GREEN	AC23	M6	GREEN
M04	AC23M6SEED	AC23	M6	WHITE
M05	AC23M6SEED	AC23	M6	WHITE
M06	AC23M6SEED	AC23	M6	WHITE
M07	AC23M7GREEN	AC23	M7	GREEN
M08	AC23M7GREEN	AC23	M7	GREEN
M09	AC23M7GREEN	AC23	M7	GREEN
M10	AC23M7SEED	AC23	M7	WHITE
M11	AC23M7SEED	AC23	M7	WHITE
M12	AC23M7SEED	AC23	M7	WHITE
M13	AC23M8GREEN	AC23	M8	GREEN
M14	AC23M8GREEN	AC23	M8	GREEN
M15	AC23M8GREEN	AC23	M8	GREEN
M16	AC23M8SEED	AC23	M8	WHITE
M17	AC23M8SEED	AC23	M8	WHITE
M18	AC23M8SEED	AC23	M8	WHITE
M19	AC85M6GREEN	AC85	M6	GREEN
M20	AC85M6GREEN	AC85	M6	GREEN
M21	AC85M6GREEN	AC85	M6	GREEN
M22	AC85M6SEED	AC85	M6	WHITE
M23	AC85M6SEED	AC85	M6	WHITE
M24	AC85M6SEED	AC85	M6	WHITE
M25	AC85M7GREEN	AC85	M7	GREEN
M26	AC85M7GREEN	AC85	M7	GREEN
M27	AC85M7GREEN	AC85	M7	GREEN
M28	AC85M7SEED	AC85	M7	WHITE
M29	AC85M7SEED	AC85	M7	WHITE
M30	AC85M7SEED	AC85	M7	WHITE
M31	AC85M8GREEN	AC85	M8	GREEN
M32	AC85M8GREEN	AC85	M8	GREEN
M33	AC85M8GREEN	AC85	M8	GREEN
M34	AC85M8SEED	AC85	M8	WHITE
M35	AC85M8SEED	AC85	M8	WHITE
M36	AC85M8SEED	AC85	M8	WHITE

Raw sequencing reads were assessed for quality with FastQC v0.12.0 [27]. The sequence adapters and low-quality reads (Phred score < 30) were trimmed with the BBDuk function with default parameters in BBTools [27]. The alignment of processed sequencing reads were executed using default parameters in HISAT2 v2.0.5 [28]. Two publicly available vanilla genomes (Daphna’ (PRJNA633886) and CR00040 (PRJNA753216)) were used as reference to assess the mapping qualities. The number of reads mapped to each gene were summarized from alignment files and counted using featureCounts v1.5.0 [29].

### Gene differential expression analysis

Differentially expressed genes (DEGs) were identified by analyzing transcript counts using DESeq2 R package with adjusted p-value 0.05 [30]. Pairwise comparisons were performed between the two accessions, two tissue types, and across the three time points to identify differentially expressed genes associated with vanillin biosynthesis. Sequences of significant DEGs were blasted against the National Center for Biotechnology Information (NCBI) non-redundant (NR) database with BLAST+ [31], and annotated with protein domains and families database from European Bioinformatics Institute (EBI) with InterProScan [32]. Gene Ontology (GO) terms were assigned to all DEGs based on the biological process, molecular function and cellular component categories using the Blast2GO v5.2.5 [33].

### qPCR expression profiling

Five of the DEGs identified in the differential expression analysis were selected for expression validation using qPCR. These genes were selected based on the occurrence from multiple comparison and their relevance to vanilla biosynthesis pathway. The expression patterns of the genes in the two vanilla accessions (AC23, low vanillin content; AC85, high vanillin content) across tissue type and developmental stages were compared. Exonic regions of the target genes were identified using Geneious software, and primers were designed with Primer3, ensuring optimal length (18–24 bases), GC content (40–60%), and melting temperatures (50–60 °C). Actin was used as the endogenous control gene. The selected genes for validation were: CPLP (*VANPL_A_00005g017160*), CPLP inh (*VANPL_A_00003g003010*), CSE (*VANPL_A_00007g009390*), CYP (*VANPL_A_00013g003220* and *VANPL_A_00001g006190*), and PAL (*VANPL_A_00003g007020* and *VANPL_A_00003g007030*). Relative expression was calculated using the ΔΔCt method, and the expression ratio (RQ) was determined as 2^−ΔΔCt^. Three replicates for each condition were used across accessions, tissue types, and developmental stages.

### GO analysis and KEGG pathway

The function of each DEG was classified using GO annotation and the enrichment analysis of the total GO pattern was performed to capture gene function distribution and metabolic pathway relations. Gene counts for each GO term were generated in R and the top ten GO terms were selected for each contrast, generating a list of the DEGs included in the most abundant GO terms. The BlastKOALA tool [34] from KEGG was used to blast the DEG sequences against eukaryote genes and assign KEGG Orthology (KO) terms to each DEG. KO assigned genes were used for pathway enrichment to single out vanillin biosynthesis related genes.

## Results

### Vanillin content evaluation for transcriptome study

The initial vanillin evaluation indicated both glucovanillin and vanillin abundance were highly variable among the seven accessions and at different time points (Supplementary Figure S2). The abundance of glucovanillin and vanillin in the seven accessions showed different progressive trends at the three sampled points corresponding to different developmental stages. Even though both AC113 and AC85 showed the highest glucovanillin abundance but only AC85 had the highest vanillin abundance at 8 MAP. Whereas AC23 has medium glucovanillin abundance but lowest vanillin abundance at 8 MAP. The dynamic changes in vanillin abundance across developmental stages highlight the complexity of vanillin biosynthesis. Since the primary breeding focus is the total vanillin, accessions AC85 and AC23 were selected for further quantitative analysis.

**Fig. 2:**
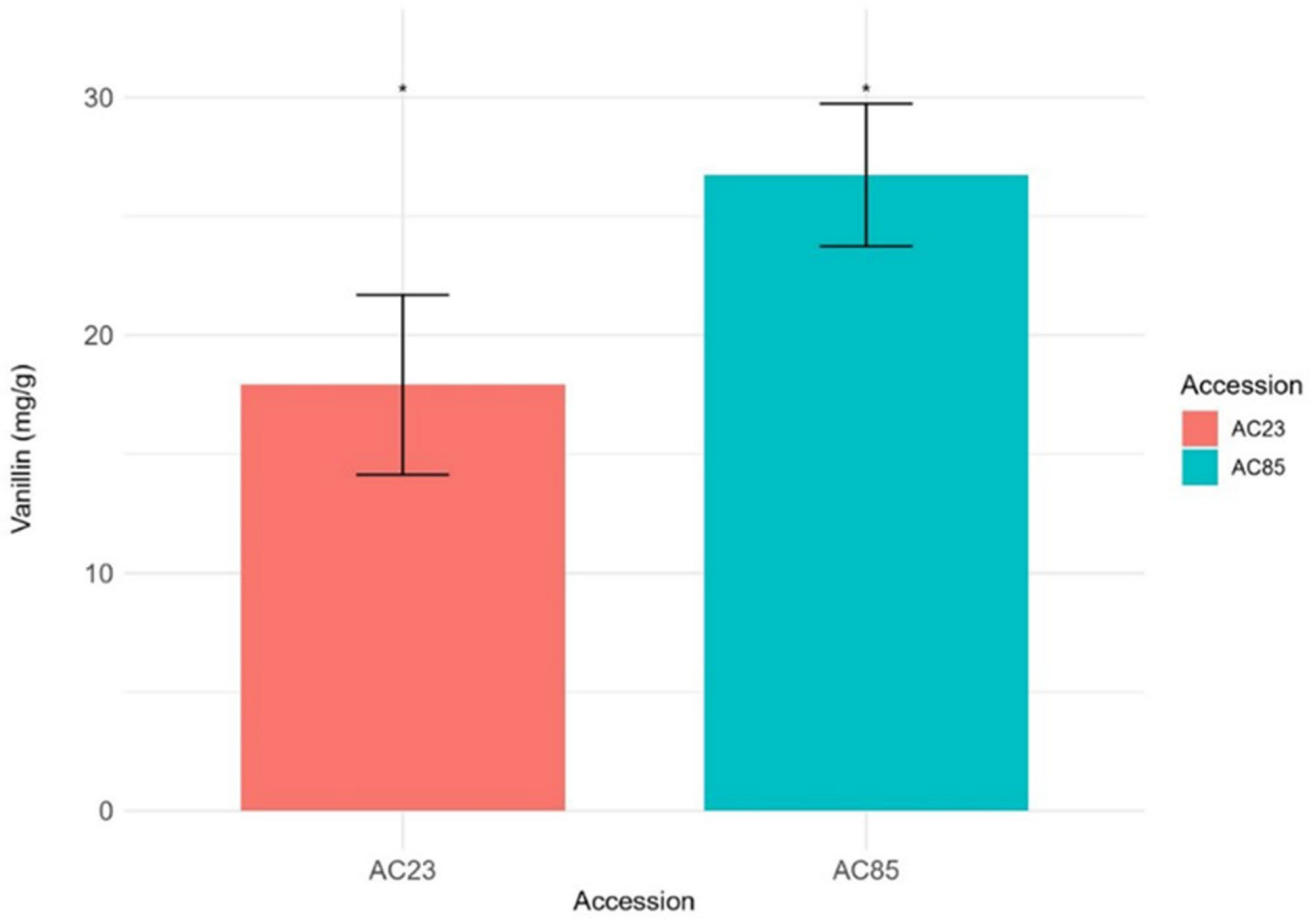
Vanillin content of AC85 and AC23 selected for transcriptome study. The absolute quantification was replicated three times for each accession. The vanillin concentration differences were found to be statistically significant (*p* < 0.05)

Further confirmation of vanillin content using LC-MS/MS revealed that AC85 initially had higher glucovanillin levels than AC23 at 6 MAP, but the levels dropped significantly and became equal by 8 MAP (Supplementary Figure S3). Conversely, vanillin content remained consistently higher in AC85 compared to AC23 throughout the three-month period. Both AC85 and AC23 reached their highest vanillin content at 8 MAP, with a statistically significant difference (*p* < 0.05) (Fig. 2). These two accessions were selected for downstream transcriptome analyses to identify candidate genes involved in vanillin biosynthesis that contribute to the differences in vanillin content.

### Transcriptome sequencing and analyses

**Fig. 3:**
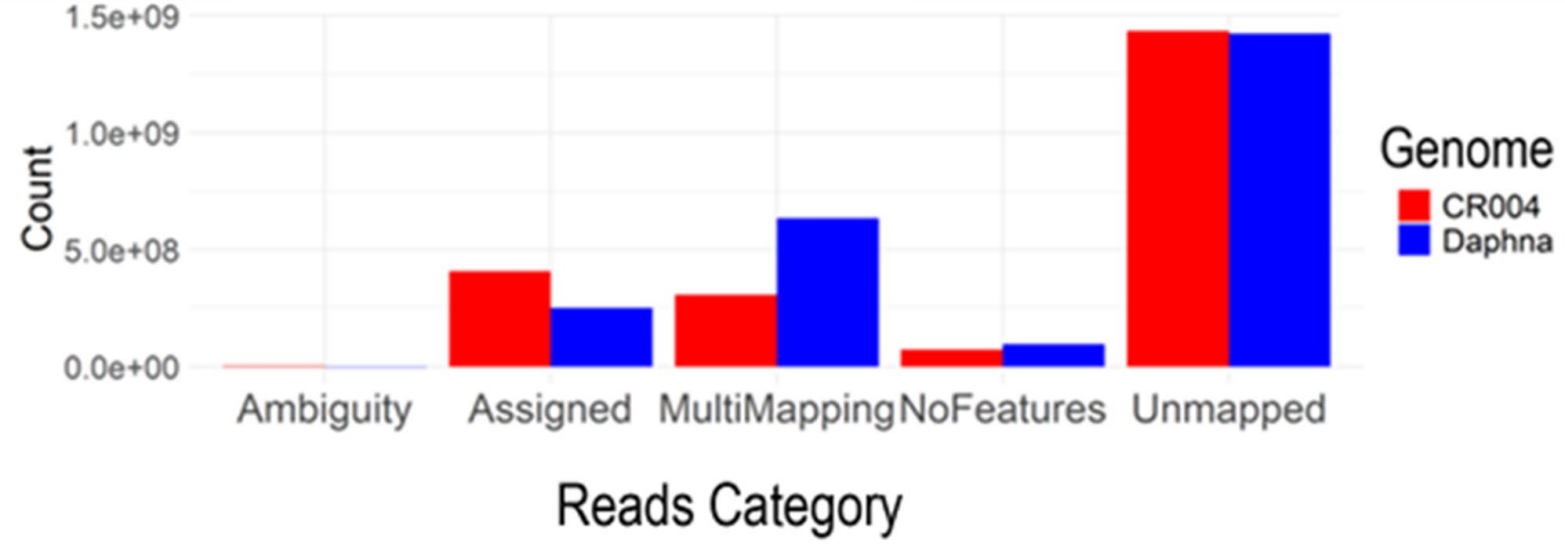
Mapping rates of transcriptome sequencing reads in two reference genomes

A total of 93.2 GB sequence reads was generated from the Illumina sequencing platform. Reads with undetermined calls higher than 10% were removed, and high-quality reads were defined as those with a Phred score of Q30 or higher, corresponding to a base call accuracy of 99%. The filtering process yielded 2,227,360,964 high quality raw reads for downstream mapping, and a total of 791,705,126 reads were mapped to the two reference genomes. The number of reads mapped to the reference genomes was 346,612,295 for Daphna and 483,854,855 for CR004, respectively. There were more multimapping reads in the Daphna genome than CR004 but the overall unmapped reads were similar (Fig. 3). The number of transcripts assigned from read counts differed significantly between the two reference genomes, with nearly twice as many transcripts identified using the CR004 genome compared to the Daphna genome. This is expected as the CR004 genome contains both haplotypes, while the Daphna genome only represents the primary haplotype. Sequencing reads were deposited at NCBI under BioProject PRJNA974693.

**Fig. 4:**
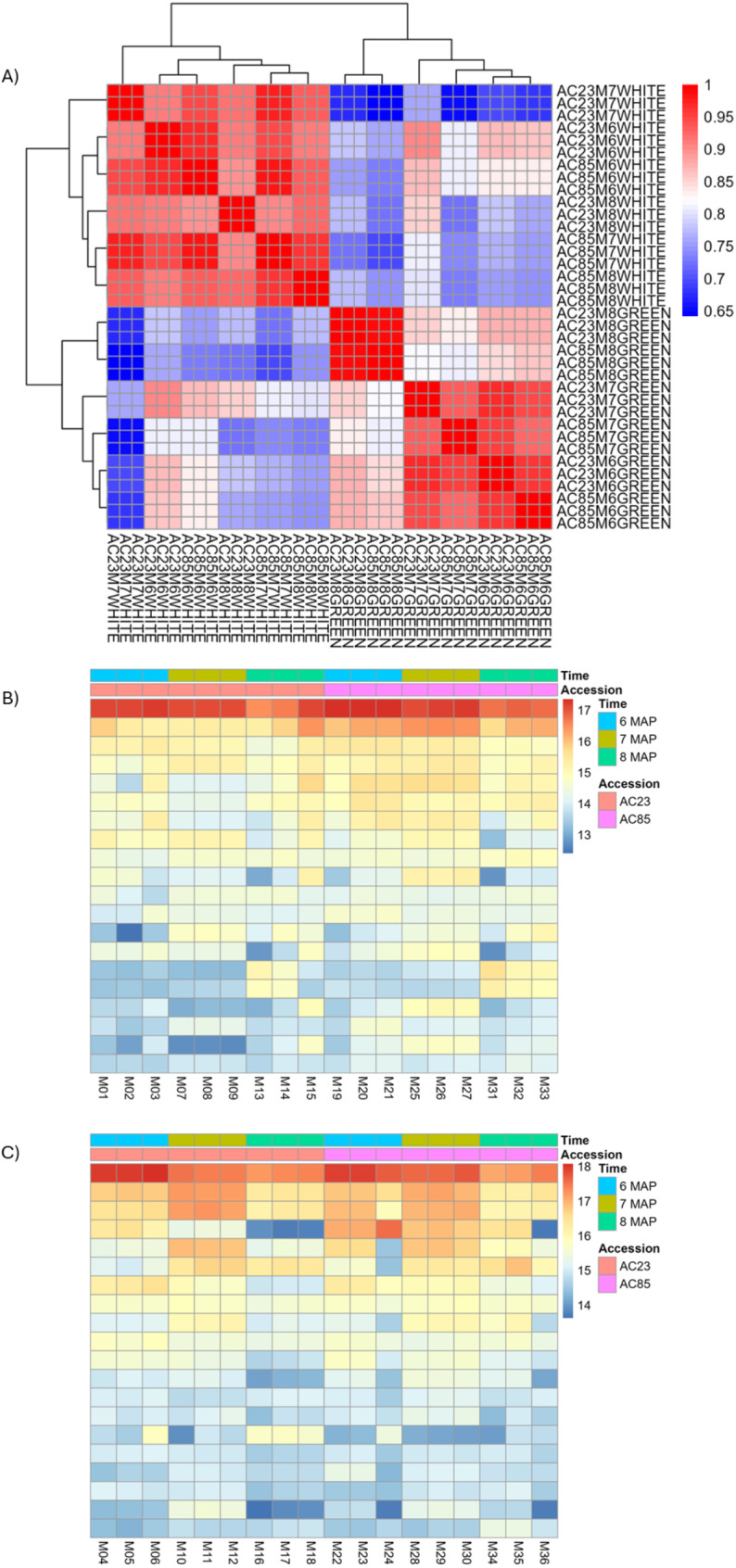
Correlation of the sequence reads obtained from individual samples. (**A**) Hierarchical cluster heatmap. Sample name contains accession code (AC23 or AC85) followed by the harvest time (6 MAP, 7 MAP and 8 MAP) with sampled tissue types (WHITE or GREEN) and the biological replicates (e.g. AC23M6GREEN1). (**B**) Heatmap for samples in the outer green tissue type. (**C**) Heatmap for samples in the inner white tissue type

The raw reads clustered around a p-value of 0 when the cutoff for the DEGs was set to 0.05 and a log2 fold change (LFC) of 0.58. The unmapped reads were identified as viral RNA contamination according to NCBI blast and subsequently removed from downstream analysis. Correlation analyses were conducted to validate the individual sample sequence quality (Fig. 4A). The two accessions and their respective tissue types showed high correlation, indicating the high quality of the individual samples and their read counts.

### DEGs and annotations

**Fig. 5:**
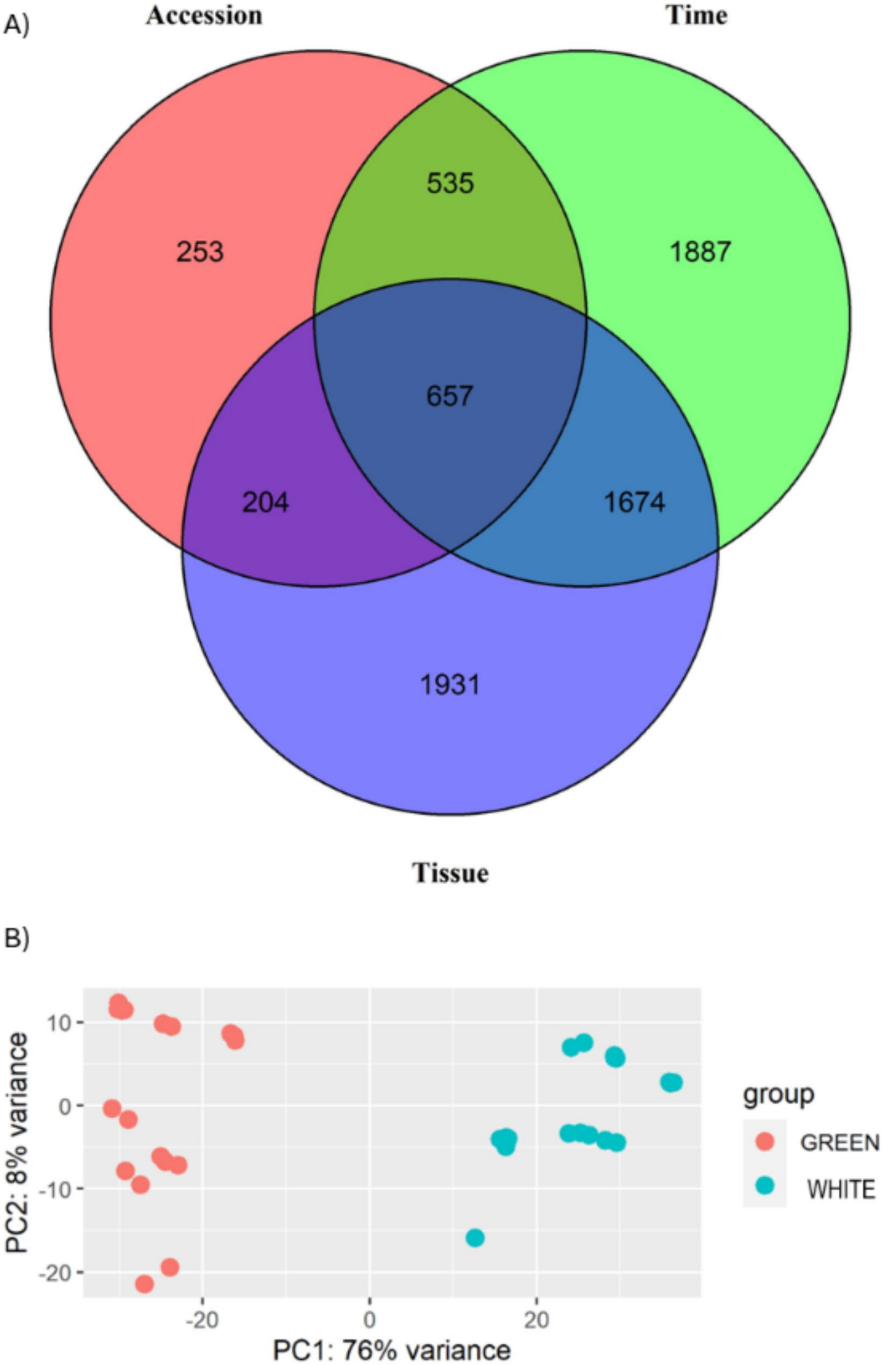
Analysis of differentially expressed genes identified from the comparisons. (**A**) Venn diagram demonstrating overlapping and unique genes. (**B**) Principal component analysis of the two tissue types

A total of 15,896 genes were identified to be differentially expressed from the three comparisons (LFC > 0.58, padj < 0.05). Among them, 2,037 differentially expressed genes were identified between the two accessions, 6,480 between the tissue types, and 7,379 DEGs were found across different time points for each accession. A total of 657 DEGs were common across all comparisons, with 535 shared between accession and time, 204 between accession and tissue, and 1,674 shared between time and tissue (Fig. 5A).

The principal component analyses were performed using the DEGs identified from the three comparisons. Genes from the two tissue types (green tissue and green tissue) of the capsules largely contributed to the clustering with the first two components explained 84% of the total variance, indicating different expression profiles of those DEGs in the outer (green tissue: epicarp) and inner layer (white tissue: mesocarp, endocarp, placental and seed tissue) of the capsule (Fig. 5B). Significant variation was identified between the two accessions in green and white tissue type, with the first two components explaining 72% and 64% of the total variance, respectively. However, a more distinct pattern of variation was observed in the green tissue (Supplementary Figures S3). The expression levels of DEGs were similar at 6 MAP and 8 MAP in both green and white tissues across the two accessions, whereas significant variation was observed at 7 MAP in both tissues between the accessions (Supplementary Figures S4).

**Fig. 6:**
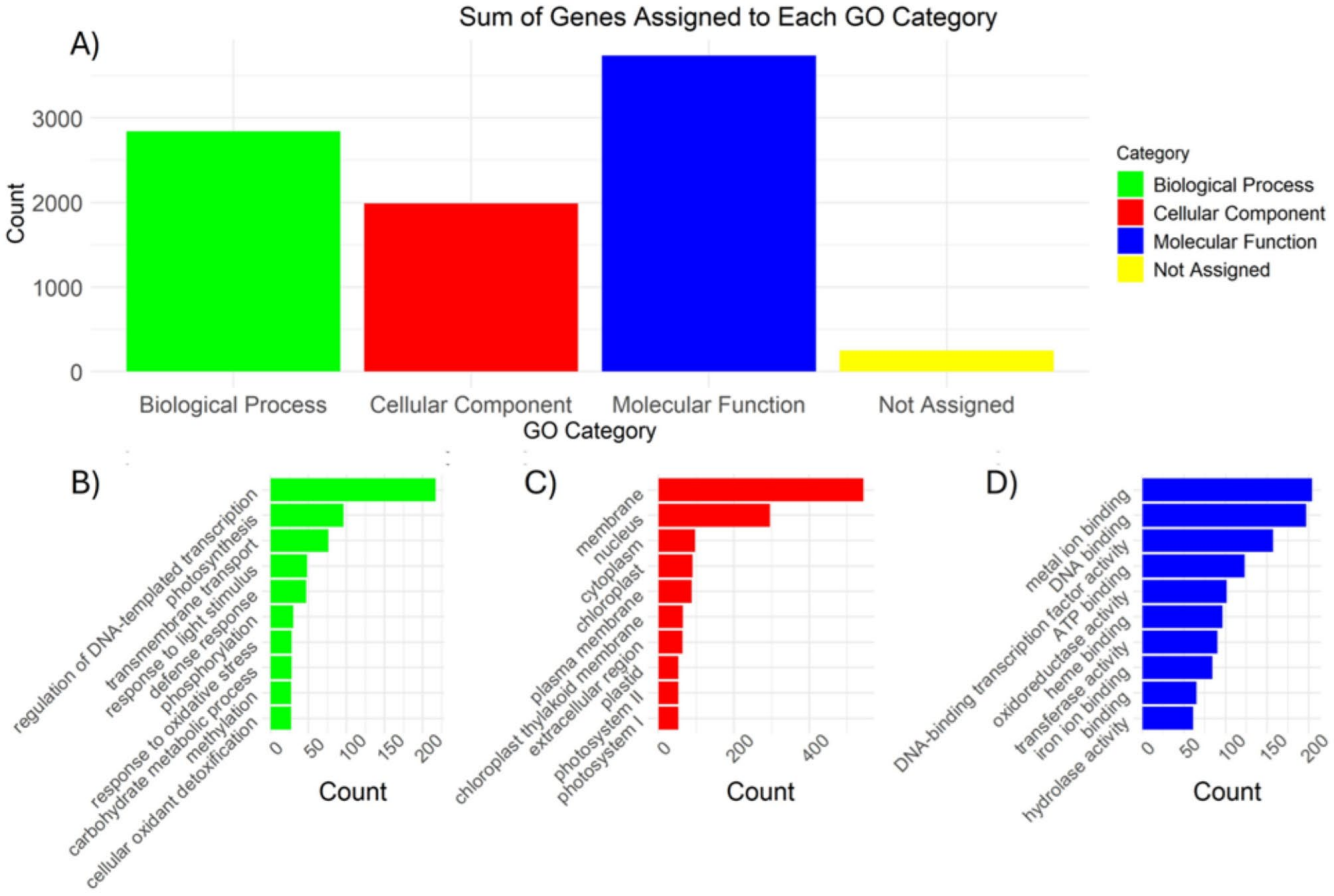
Gene ontology analysis of commonly shared differentially expressed genes (DEGs). (**A**) Distribution of DEGs amongst GO categories. (**B**) Top 10 GO terms in Biological Process. (**C**) Top ten GO terms in Cellular Component. (**D**) Top ten GO terms in Molecular Function

**Fig. 7:**
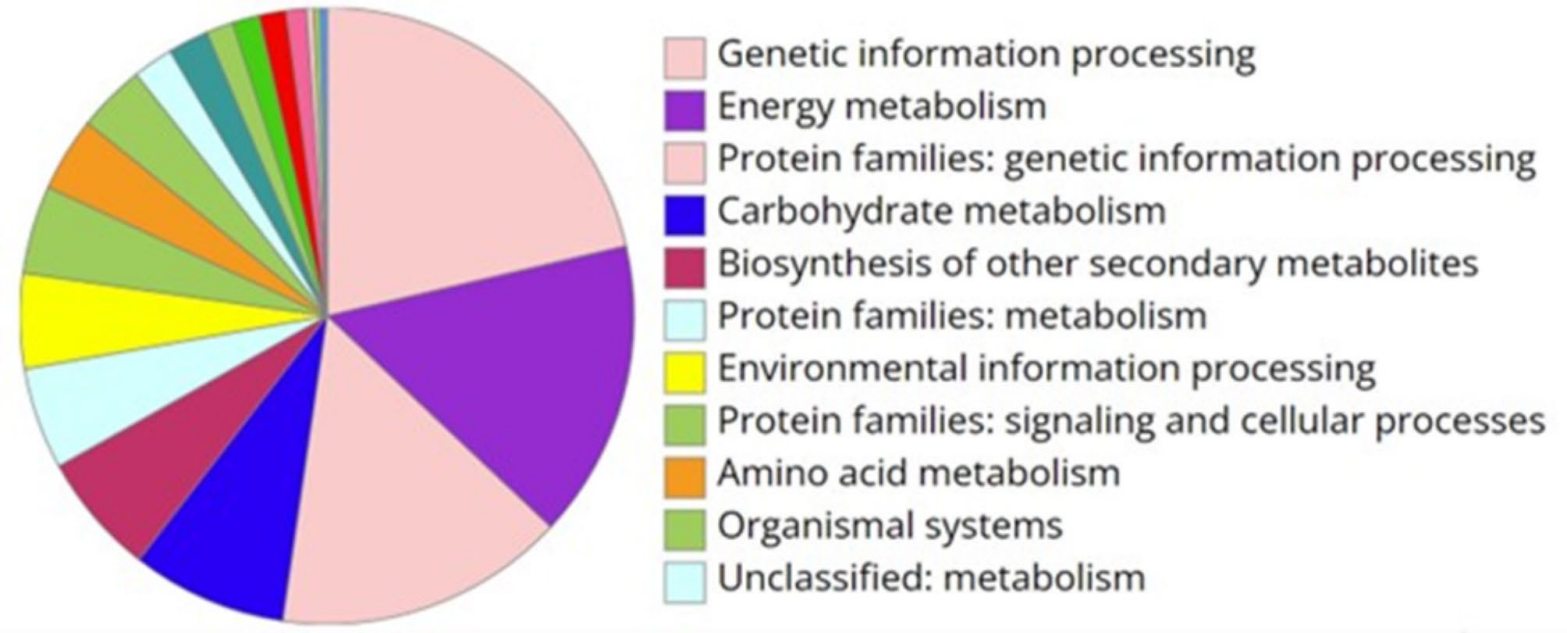
KEEG pathway enrichment analysis results showing DEG distribution amongst the KO terms

A total of 2,448 highly differentiated genes were identified from the three comparisons with the cut-off value was set at LFC ≥ 2 and padj < 0.01. The Gene Ontology (GO) analysis revealed that most DEGs were assigned to molecular functions (45.8%) followed by biological processes (30.7%), and cellular components (23.5%) (Fig. 6). These genes were then subjected to pathway enrichment analysis, which assigned KO terms and identified 281 gene associated with the biosynthesis of other secondary metabolites (Fig. 7).

The comparison between the accessions revealed 161 DEGs, with ten of them involved in vanillin biosynthesis (Supplementary Table S1). These genes included cytochrome P450 (CYP), *O-*methyltransferase (OMT), cysteine-rich and transmembrane domain-containing protein, β-glucosidase (β-Glu), and PAL. Multiple members of the same gene families having similar putative functions were detected, showing different expression patterns among them. For example, three genes (*VANPL_A_00002g010340*, *VANPL_A_00008g003200* and *VANPL_A_00004g005220*) were found to code for homologous of CYP, while the additional three genes (*VANPL_A_00014g004350*, *VANPL_A_00014g003980* and *VANPL_A_00014g004010*) were homologous of caffeic acid 3-*O*-methyltransferase. The low vanillin accession (AC23) had 12 DEGs in the inner white tissue between 6 MAP and 7 MAP when compared to the high vanillin accession (AC85). Of them, two genes were downregulated and the rest were upregulated. One of the gene was *VANPL_00513g000160*, encoding 4-hydroxy-4-methyl-2-oxoglutarate aldolase. This enzyme is involved in the synthesis of pyruvate, a source of phosphoenol pyruvate (PEP) and precursor of shikimic acid, which both have been reported to play a role in pathways closely related to vanillin biosynthesis [35]. A total of 1,534 genes were found highly differentially expressed (LFC > 2, padj < 0.01) between the tissue types of the two accessions (Supplementary Table S2). Among these genes, 42 codes enzymes such as glucosyltransferase, β-Glu, CoA synthase, 4-coumarate-CoA ligase, PAL, cysteine-rich receptor protein, *O*-hydroxycinnamoyl transferase, CYP and *O*-methyl transferase. Among the 753 genes identified as differentially expressed in the time comparison, 25 genes are directly involved in at least one step of the vanillin biosynthetic pathway (Supplementary Table S3). The most significant difference was observed between 7 MAP and 8 MAP, suggesting this period is critical for vanillin accumulation. The enzymatic functions identified include *O*-glucosyltransferase, cysteine-rich receptor protein kinase, 4-coumarate–CoA ligase, CYP, PAL, short-chain dehydrogenase reductase, caffeoyl shikimate esterase (CSE), CoA synthase, OMT and omega-hydroxypalmitate *O*-feruloyl transferase.

Genes that were recurrently highlighted across multiple comparisons warranted closer analyses. The *VANPL_A_00014g004010* was found to be significantly expressed in three comparisons. This gene encodes caffeic acid 3-*O*-methyltransferase, an important enzyme for the conversion of caffeic acid into ferulic acid which is one of the steps considered in the proposed ferulate vanillin biosynthetic route [18, 24, 36]. Three genes (*VANPL_A_00003g007020*, *VANPL_A_00014g003990*, and *VANPL_A_00014g003980*) were differentially expressed in both accession and time comparisons. Of them, *VANPL_A_00003g007020* encodes PAL, an important enzyme that serves as the initial step of vanillin synthesis [22, 37, 38]. Additionally, both *VANPL_A_00014g004010* and *VANPL_A_00014g003990* encode for different variants of caffeic acid 3-*O*-methyltransferase, and both were upregulated in the high vanillin accession. This suggests a positive regulatory effect shifting metabolic activities towards the vanillin biosynthetic pathway. The *VANPL_A_00003g001090*, which encodes a β-glucosidases enzyme, was significantly upregulated at 8 MAP. Notably, this enzyme was mostly present in the outer green tissue of the capsule, which agrees with findings from previous studies [39]. Two genes directly involved in the vanillin biosynthetic pathway were identified in both accession and tissue comparisons. The *VANPL_A_00014g004350* encodes OMT and the *VANPL_A_00008g003200* encodes CYP. Eight additional genes were found to be differentially expressed in both the tissue and time comparisons. Two of them (*VANPL_A_00012g007840* and *VANPL_A_00003g006690*) code subfamilies of CYP (CYP94C and cytochrome CYP77A). The *VANPL_A_00007g009390* encodes CSE, an enzyme involved in the lignin biosynthetic pathway that hydrolyzes caffeoyl shikimate [40]. The *VANPL_A_00014g005560* encodes a type II acetyl-CoA C-acyltransferase which is associated with the mevalonate pathway in the biosynthetic direction [41]. The *VANPL_A_00002g003090* encodes an *O*-glucosyl transferase and was found to be upregulated in the white tissue as well as at 8 MAP. Lastly, hydroxycinnamoyl transferase, encoded by *VANPL_A_00011g006410*, was upregulated in the white tissue but downregulated at 8 MAP.

### Vanillin biosynthesis pathways

**Fig. 8:**
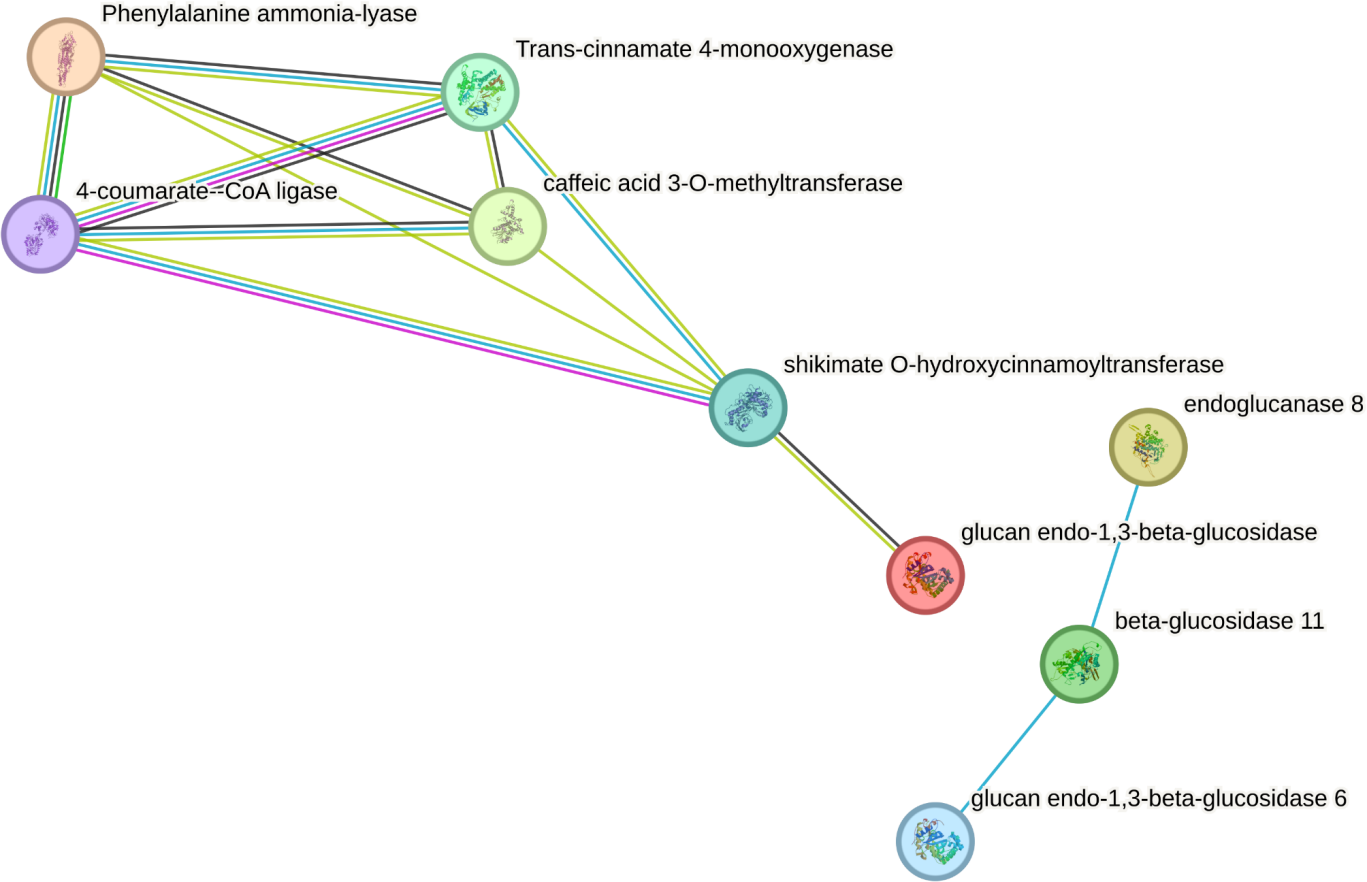
String diagram of nine proteins derived from thirteen differentially expressed genes in the phenylpropanoid pathway and the sugar sucrose metabolic pathway

A total of 64 highly differentially expressed genes (Supplementary Table S4) encoding 50 distinct protein functions associated with vanillin biosynthesis were used to reconstruct the vanillin biosynthesis pathway. The KO terms for the proteins were imputed in the “Reconstruct Pathway” tool in KEGG to visualize the involvement of the 50 proteins of interest in the vanillin metabolic pathways. Eleven of the proteins from the biosynthesis of secondary metabolites, six in the phenylpropanoid biosynthesis pathway, and four related to the starch and sucrose metabolism were highlighted. By using the sequences of the six enzymes involved in the phenylpropanoid pathway, along with the three involved with the starch and sucrose metabolism, a string diagram was generated based on the protein-protein interactions, gene fusion, co-occurrence and gene neighbor joining that clustered the proteins to their respective pathways (Fig. 8).

### Transcription expression validation

**Fig. 9:**
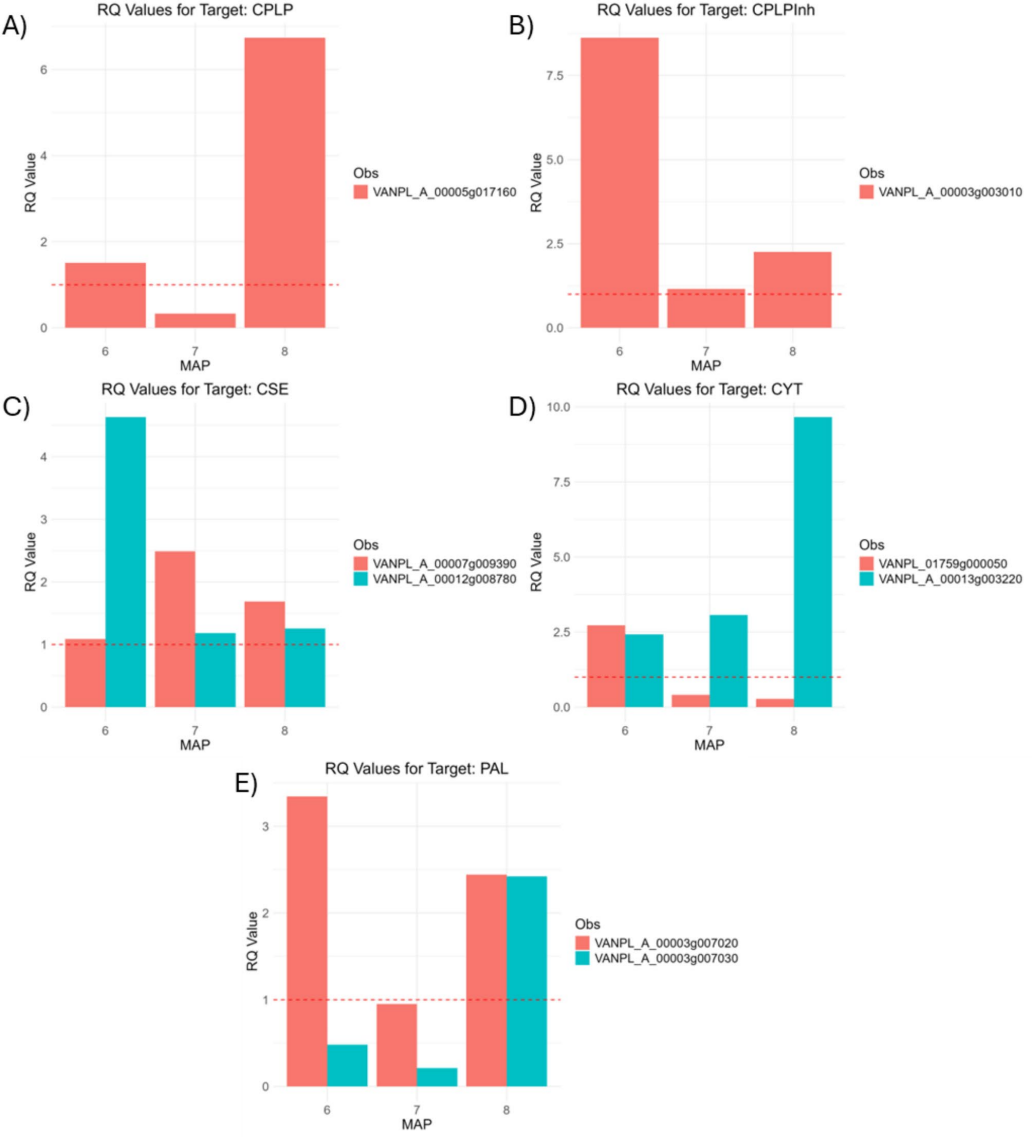
Validation of transcription patterns using relative abundance (RQ) calculated with qPCR. The Y-axis shows the RQ, with values above the red dotted line (> 1) indicating higher expression in genotype AC85 compared to AC23 (reference). The X-axis represents months after pollination (MAP). Panels: **A**) VANPL_A_00005g017160, **B**) VANPL_A_00003g003010, **C**) VANPL_A_00007g009390 and VANPL_A_00012g008780, **D**) VANPL_A_00013g003220 and VANPL_01759g000050, **E**) VANPL_A_00003g007020 and VANPL_A_00003g007030, all measured in the white tissue

The transcription expression patterns observed with the qPCR revealed specific differences between the genotypes at specific time points of the capsule development (Supplementary Table S5). For instance, the CPLP gene (*VANPL_A_00005g017160*) had a higher relative expression in AC85 than AC23 at months six and eight after pollination but not at month seven where it presented a lower relative expression compared to AC23. In the case for the CPLP inhibitor gene (*VANPL_A_00003g003010*) the gene always had a higher relative expression in AC85 but the expression ration was highest at month six. The qPCR expression profile of the validation genes correlated with the findings of the transcriptome study (Fig. 9).

## Discussion

Vanilla is considered the most popular flavor in the world and is commonly used by the food and perfume industry. For historical reasons, vanilla cultivation has been limited to a certain geographic region distant from its center of origin. Advancements in tissue culture have increased germplasm availability while reducing wild orchid populations vulnerability, including vanilla, as internationally recognized endangered species. Recent genetic studies have revealed a great potential for the genetic improvement of vanilla in the U.S, Mexico, Madagascar and Reunion [13, 42–44]. Though thousands of molecular markers have been identified for vanilla, phenotype evaluation of important traits, such as yield and aromatic compounds, are much needed for modern vanilla breeding programs. Vanillin is the major and most abundant compound in natural vanilla extract. It is also one of the traits used to grade vanilla cured capsules [5]. The phenotypic evaluation of vanillin content among the vanilla accessions in this study indicated high variability of this trait presenting the potential of improving vanillin content through breeding. Additional breeding tools such as molecular markers are crucial to improve the selection efficiency given the long juvenile period of vanilla plants.

In commercial production, vanilla capsules are harvested between eight and nine months after pollination upon maturity [10]. Van Dyk et al. (2014) identified that glucovanillin accumulating in the capsule initiated at five months after pollination, with continuous increase until month ten in a “more or less” linear manner. Although the 5 MAP stage was not included as a sampling point in this study, the 7 MAP stage emerged as a critical turning point for glucovanillin accumulation, as demonstrated by both relative abundance and quantitative analyses. This trend is further supported by gene expression profiling, which revealed a higher number of differentially expressed genes (DEGs) at seven months compared to the other time points in both accessions (Supplementary Table S3). These findings are valuable for optimizing vanilla cultivation management, emphasizing the importance of critical care at this stage. Moreover, this insight provides important information in identifying candidate genes associated with variations in vanillin content across different germplasm, as these genes may play a more significant role.

**Fig. 10:**
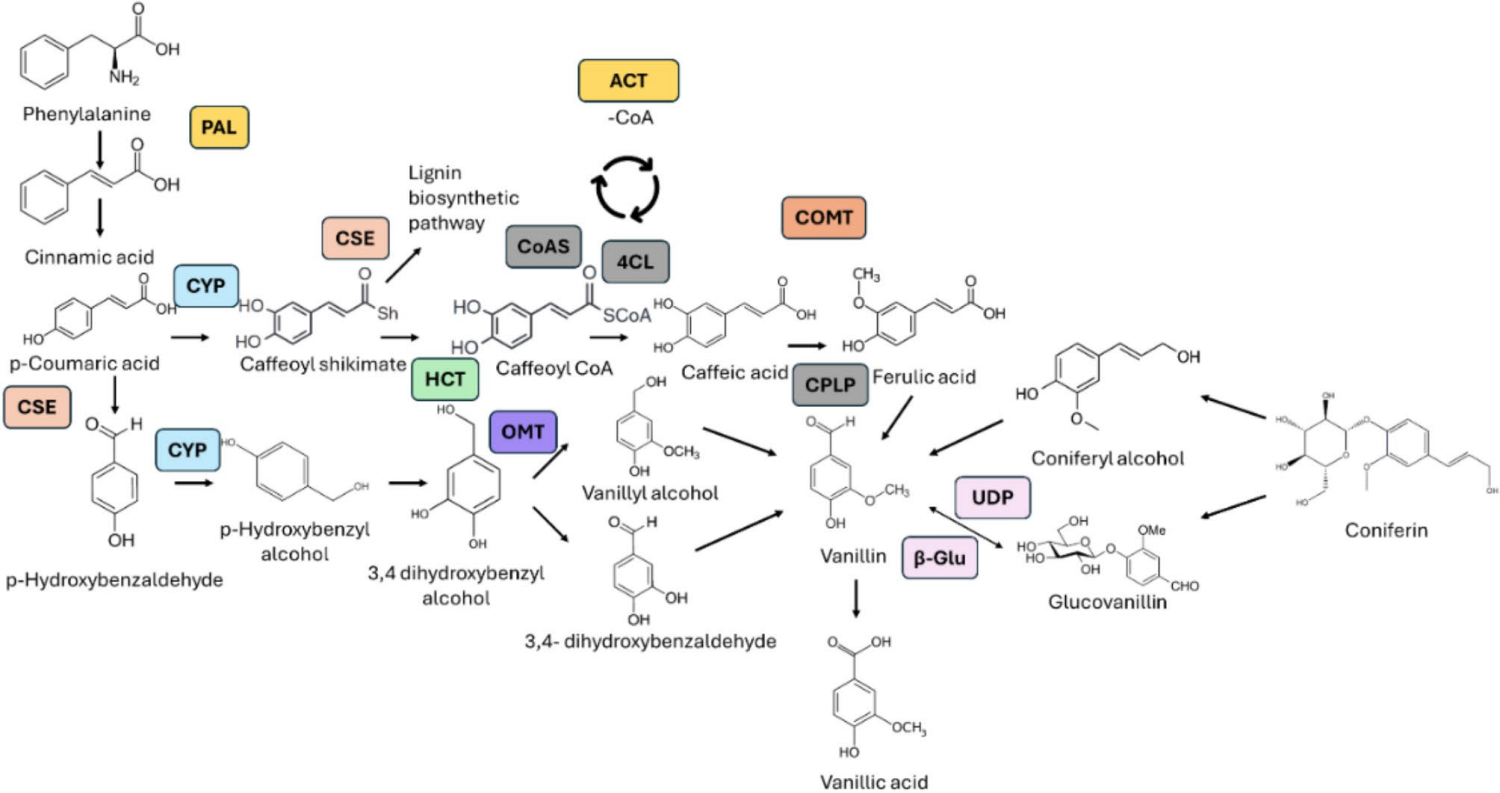
Vanillin biosynthetic pathway with highlighted enzymes found from transcriptomic DEG analysis

Differentially expressed genes identified between the accessions with contrasting vanillin content represent potential genomic variations that effect the final vanillin levels in vanilla. The DEGs identified from different genetic backgrounds were involved in multiple intermediate steps of the vanillin biosynthetic pathway. These genes encode enzymes such as Cinnamate 4-hydroxylase (C4H), Hydroxycinnamoyl transferase (HCT), CoA synthetase (CS), Methyltransferase and β-Glu, all of which were overexpressed in the high vanillin content accession in this study. Meanwhile, the homologous versions of these genes or genes encoding for competing enzymes, such as CYP 77A1, SWEET14 (from the carbohydrate metabolism pathway) or 4-coumarate–CoA ligase (from the lignin biosynthetic pathway), were downregulated in the high vanillin content accession. A truncated diagram of the vanillin biosynthetic pathway was constructed using the enzymes encoded by the DEGs identified from this study (Fig. 10; Supplementary Table S4). Eight genes associated with the phenylpropanoid pathway were found to be differentially expressed in this study, highlighting significant overlap between the phenylpropanoid and the vanillin pathways. Additional DEGs were found to be involved in the starch and sucrose metabolism, as well as the lignin biosynthetic pathway. These findings align with the discovery made by Fock-Bastide et al. [23]. Glucovanillin and other phenolic compounds are stored in chloroplast-derived organelles known as phenyloplasts [45]. In this study, the DEG analysis between accessions identified 194 chloroplast-related genes that could serve as valuable targets in breeding high vanillin cultivars through molecular breeding. Previous studies primarily focused on identifying genes involved in the vanillin biosynthesis pathway, with most attention on either the initial step (PAL) or a hypothetical final step (cysteine proteinases like protein) [18, 21]. Our present study took a comparative approach to capture the genes expressed at key developmental stages of vanilla capsule, aiming to narrow down the critical genes in the vanillin biosynthesis pathway that contribute to final vanillin content. The identified genes, including HCT, acetyl-CoA C-acyltransferase, methyltransferase and β-Glu, were found to be highly expressed not only at the initial and final steps, but also at several intermediate steps of the vanillin biosynthetic pathway. These genes were consistently upregulated in high vanillin content accessions, indicating their important role in natural vanillin production.

The vanillin biosynthesis pathway is a complex and important area for studying flavor-related metabolic research in plants, yet it remains poorly understood. Previous efforts to elucidate this pathway have largely focused on bioconversion and bioengineering for vanillin production. However, it is essential to understand the genes and genetic segments that determine final vanillin content in order to breed new cultivars with superior vanillin yield for natural vanilla production. Several genes, such as CPLP and PAL, have been reported to play key roles in this pathway [12], which aligns with the findings of the current study (Supplementary Table S4). Five CPLP genes were identified across comparisons of accessions, tissue types and time points, while three PAL genes were identified between accessions and different tissue types, highlighting the functional importance of each gene copy in vanilla capsule development (Supplementary Table S1and S2). Among different gene copies, both *VANPL_A_00002g009470* (*CPLP*) and *VANPL_A_00003g007020* (*PAL*) were upregulated in high vanillin content accession AC85, indicating their critical role in determining the final vanillin level. Additionally, two caffeic acid 3-*O*-methyltransferase genes (*VANPL_A_00014g003980* and *VANPL_A_00014g003990*) were upregulated in the green tissue of the high vanillin accession AC85, and downregulated in the white tissue of low vanillin accession AC23 from 7 MAP to 8 MAP. While vanillin is generally believed to be produced in the tissues around the seeds [46], corresponding to the white tissue in this study, it is possible that the green tissue of the vanilla capsule may also contribute to the overall vanillin content as part of the C-lignin biosynthesis process. The involvement of OMT genes in determining final vanillin content is consistent with previous findings and hypothesis indicated by Rao et al. (2014), this study provides further insights in specific gene functions and locations.

Having identified the genes present in *V. planifolia* capsules, along with their locations and functions in vanillin accumulation, lays the foundations for further research into the genomic mechanisms of this biosynthesis pathway, which can facilitate the development of molecular tools to increase vanillin content through modern plant breeding. This can be achieved by identifying the genetic variations of key genes that determine vanillin production for marker development to select elite breeding lines with high vanillin content. To fully harness the genetic potential for natural vanillin production, genetic resources such as diverse plant material with a wide range of phenotypic variation, along with the genetic variation associated with them, can be leveraged through strategic crossing and selections. The *Vanilla* genus encompasses over a hundred species with significant variations in flavor profile. While many *Vanilla* species can develop capsules, not all produce aromatic compounds. The substantial flavor profile differences between species such as *V. planifolia* and *V. pompona* highlight the potential for breeding new vanilla hybrids with unique flavor profiles, as has been demonstrated in *V. x tahitensis*. The key genes discovered in this study will serve as valuable tools not only for studying the vanillin biosynthesis pathway and its evolution of aromatic metabolites across different vanilla species but also for understanding the genetic variations critical for marker development, ultimately leading to vanilla hybrids with new flavor profiles.

## Conclusions

In conclusion, the HPLC and LC MS/MS approaches were used to screen vanilla accessions with distinct vanillin content, and a comparative transcriptome study was conducted to identify key genes in the vanillin biosynthesis pathway across different accessions, tissue types and developmental stages. Using a fully annotated genome, a total of 2,448 differentially expressed genes were identified across three comparisons, with 75 genes identified in more than one comparison as being associated with vanillin biosynthesis pathway. The seven months after the pollination appears to be a critical time point for metabolites synthesis in vanilla capsules. The results indicated that vanillin accumulation in vanilla is driven by multiple biosynthesis pathways, with several copies of *CPLP*, *PAL*, and *CMOT* involved in various steps and locations of vanillin accumulation process. In addition, key genes *VANPL_A_00002g009470* (*CPLP*), *VANPL_A_00003g007020* (*PAL*), *VANPL_A_00014g003980* (*CMOT*) and *VANPL_A_00014g003990* (*CMOT*) were identified as highly importance for vanillin content variation among accessions. These genes present valuable resources for future marker-assisted breeding efforts.

## Electronic supplementary material

Below is the link to the electronic supplementary material.

Supplementary Material 1

Supplementary Material 2

## Data Availability

The files for the raw reads were submitted in the Sequence Read Archive data repository from NCBI under the BioProject: PRJNA974693 (https://www.ncbi.nlm.nih.gov/bioproject/PRJNA974693).

## Abbreviations

DEG: Differentially expressed gene
MAP: Month after pollination
LFC: Logarithmic fold change
PAL: Phenylalanine ammonia lyase
CYP: Cytochrome P450
CSE: Caffeoyl shikimate esterase
HCT: Hydroxy cinnamoyl transferase
4CL: 4-hydroxycinnamoyl-CoA ligase
OMT: Methyltransferase
β-Glu: β-glucosidase
CS: CoA synthetase
CPLP: Cysteine protease-like protein
