# Supplementary material for: Comparative transcriptome profiling of vanilla (Vanilla planifolia) capsule development provides insights of vanillin biosynthesis
Source: BMC Plant Biol. 2025 Mar 18;25:343. doi: 10.1186/s12870-025-06360-w (PMC11916913; doi:10.1186/s12870-025-06360-w)
Supplement: Supplementary file 1 — Supplementary Material 1 [file 12870_2025_6360_MOESM1_ESM.docx]

Supplemental Figures


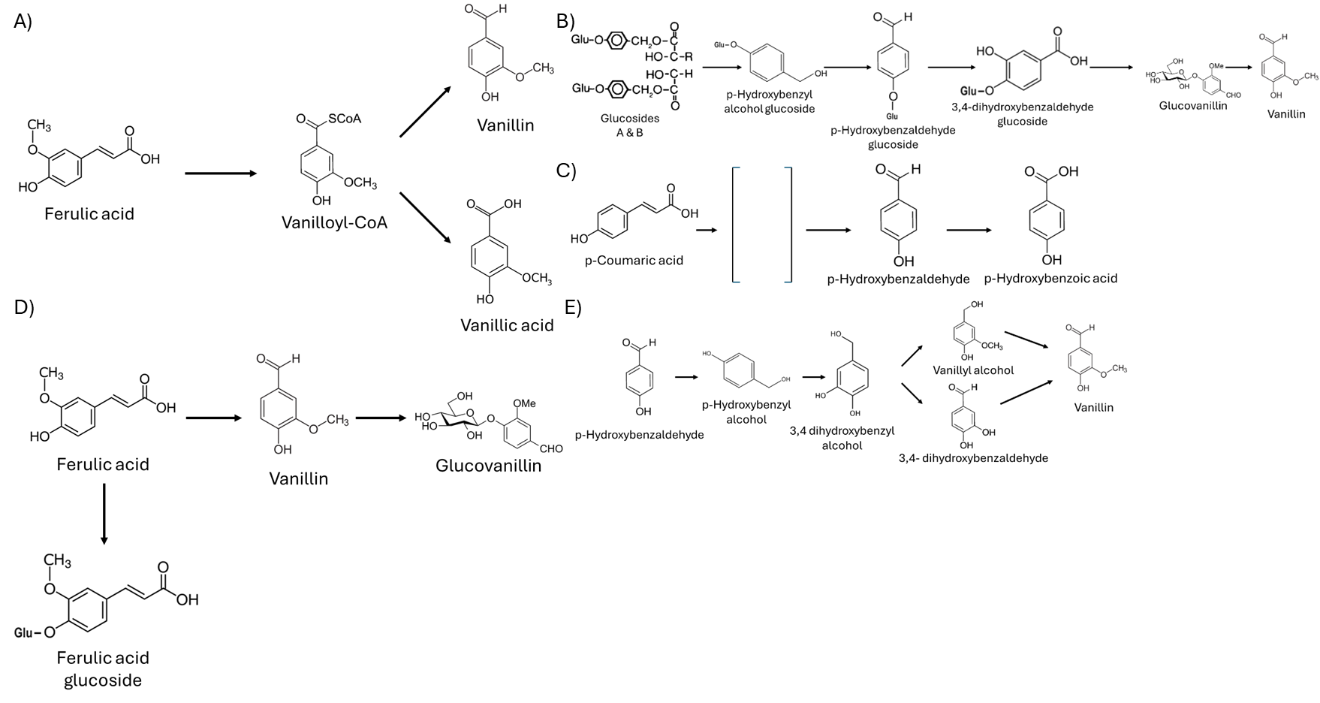


Figure S1. A) Zenk vanillin pathway (Palama, 2010; Kundu, 2017). B) Kanizawa vanillin alternative pathway with novel glucosides (Kanisawa, Tokoro and Kawahara, 1994). C) Yazaki non oxidative chain shortening pathway (Yang et al., 2017). D) Benzoate route for vanillin synthesis (Havkin-Frenkel and Belanger, 2011). F) Ferulic acid – vanillin VpVAN mediated pathway (Gallage et al., 2014).


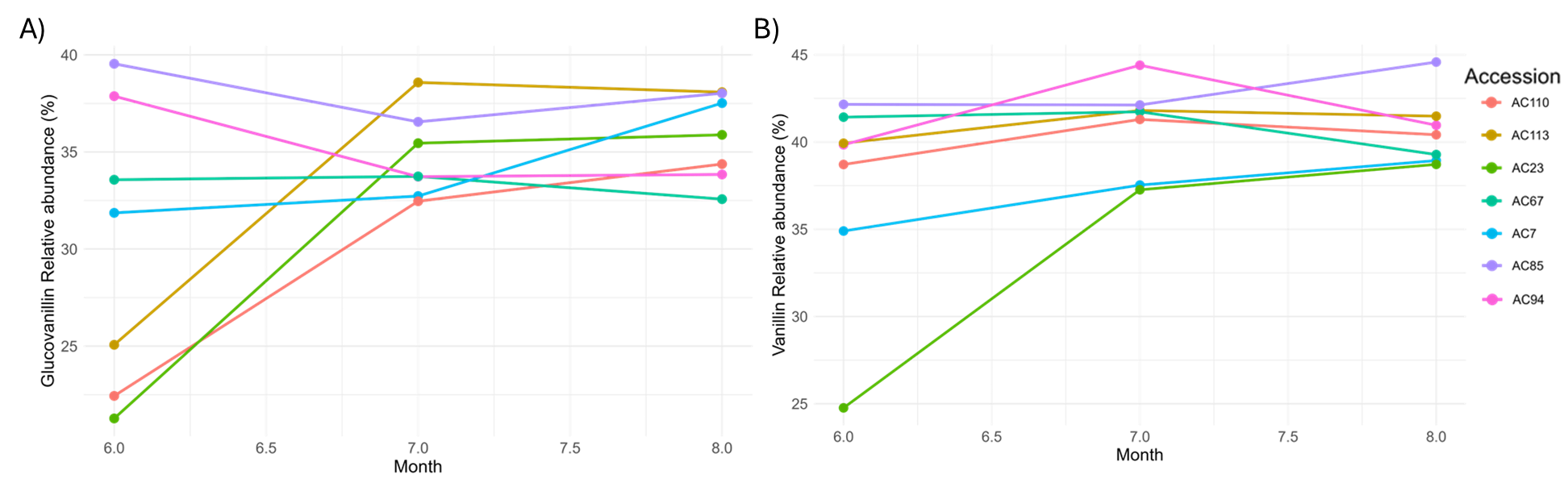


Figure S2. HPLC-based relative abundance of glucovanillin (A) and vanillin (B) in seven vanilla accessions at 6, 7 and 8 months after pollination (MAP).


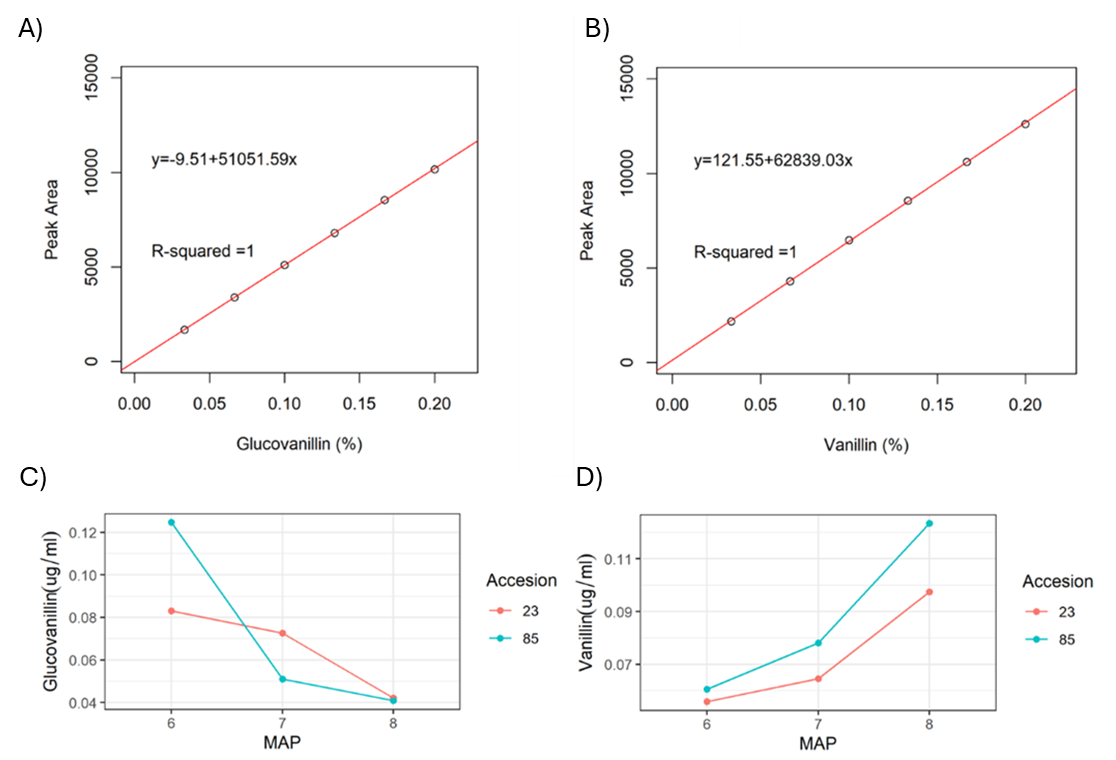


Figure S3. Vanillin and glucovanillin relative abundance of AC23 and AC85 using LC MS/MS. A) Glucovanillin calibration curve. B) Vanillin calibration curve. C) Glucovanillin relative abundance. D) Vanillin relative abundance.


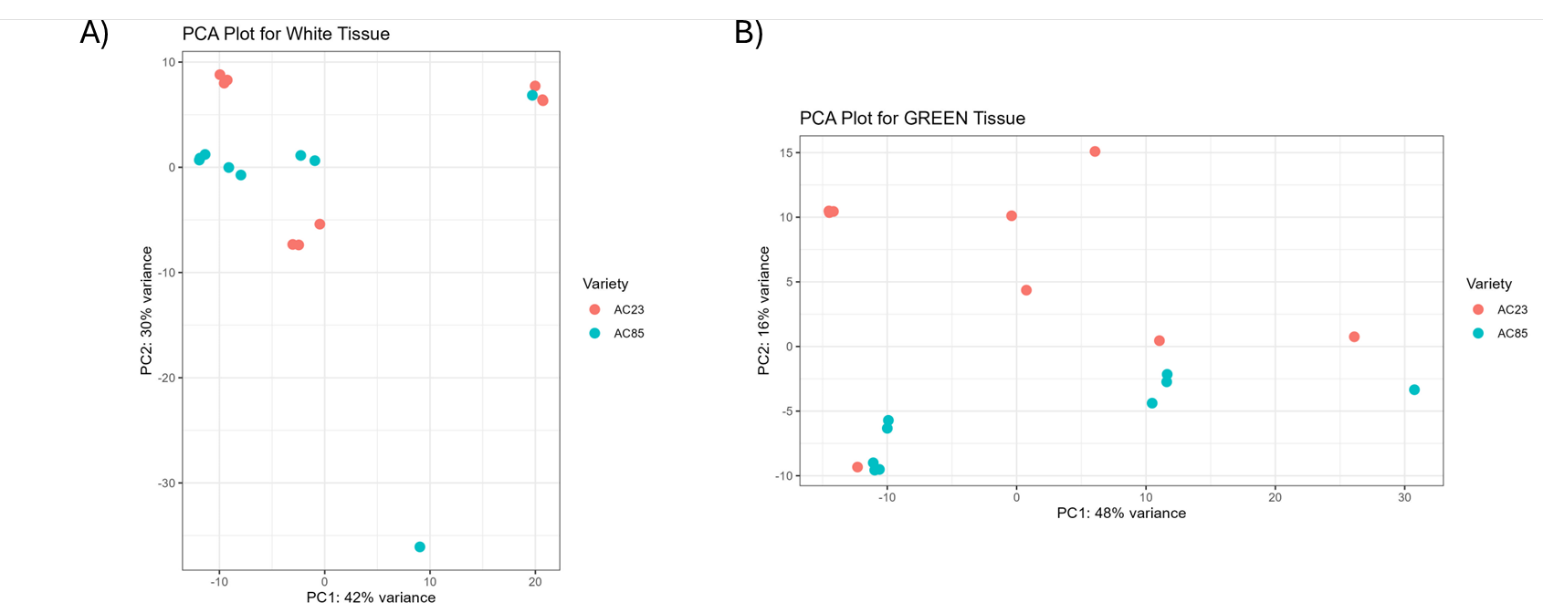


Figure S4. PCA plots for separate tissue types. A) White tissue. B) Green tissue.


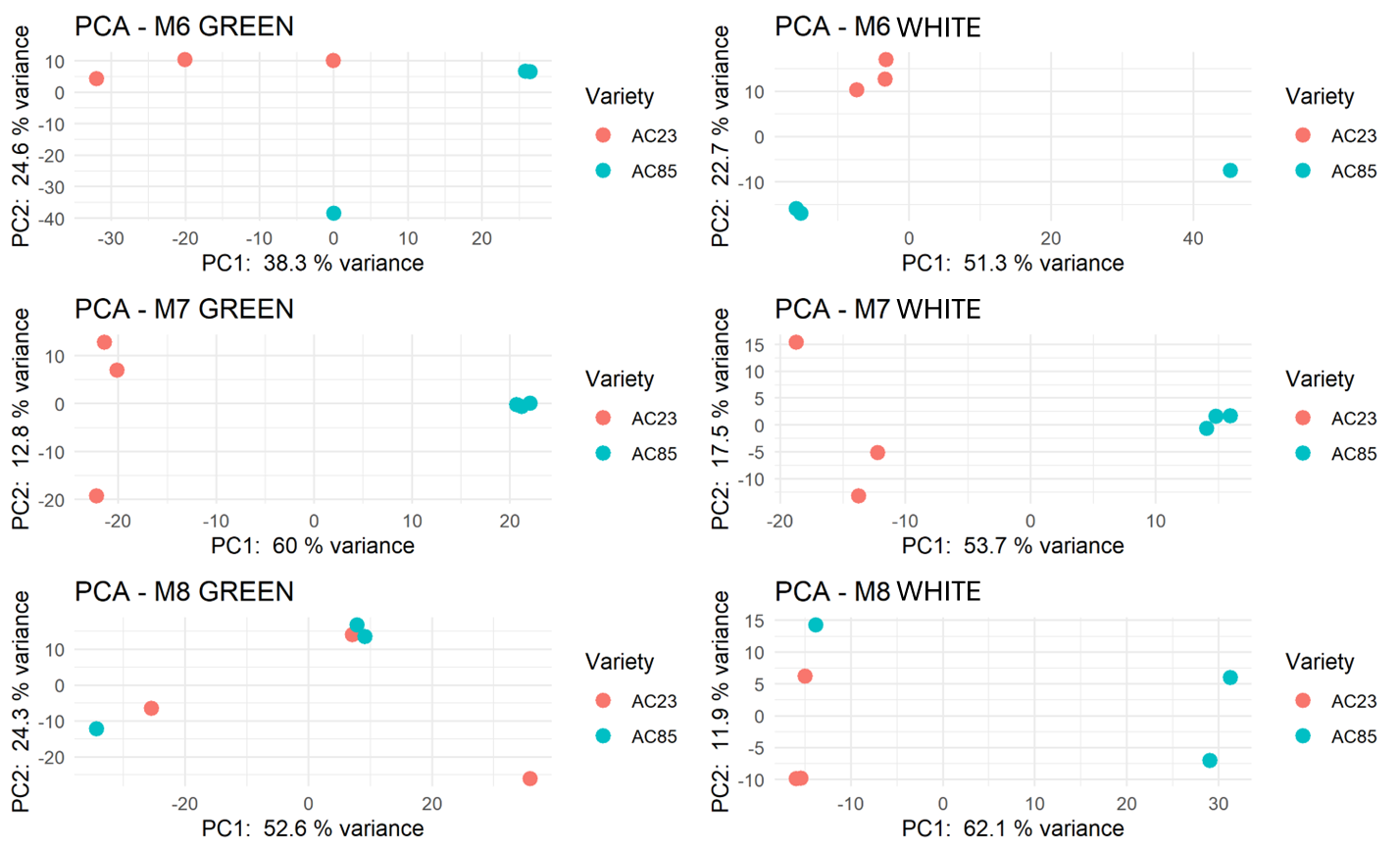
Figure S5. PCA Plots for each tissue and time point for both accessions.
